# Younger epigenetic age is associated with higher cardiorespiratory fitness in individuals with airflow limitation

**DOI:** 10.1016/j.isci.2024.110934

**Published:** 2024-09-13

**Authors:** Ana I. Hernandez Cordero, Carli Peters, Xuan Li, Chen Xi Yang, Amirthagowri Ambalavanan, Julie L. MacIsaac, Michael S. Kobor, Gregory J. Fonseca, Dany Doiron, Wan Tan, Jean Bourbeau, Dennis Jensen, Don D. Sin, Graeme J. Koelwyn, Michael K. Stickland, Qingling Duan, Janice M. Leung, the CanCOLD Collaborative Research Group

**Affiliations:** 1 Centre for Heart Lung Innovation, St. Paul’s Hospital and University of British Columbia, Vancouver, Canada; 2 Edwin S. H. Leong Healthy Aging Program, Faculty of Medicine, University of British Columbia, Vancouver, Canada; 3 Department of Biomedical and Molecular Sciences, School of Medicine, and School of Computing, Queen’s University, Kingston, Canada; 4 Centre for Molecular Medicine and Therapeutics, University of British Columbia, Vancouver, Canada; 5 McGill University Health Centre, McGill University, Montreal, Canada; 6 Clinical Exercise & Respiratory Physiology Laboratory, Department of Kinesiology and Physical Education, Faculty of Education, McGill University, Montreal, Canada; 7 Division of Respiratory Medicine, Department of Medicine, University of British Columbia, Vancouver, Canada; 8 Faculty of Health Sciences, Simon Fraser University, Burnaby, Canada; 9 Division of Pulmonary Medicine, Faculty of Medicine and Dentistry, University of Alberta, Edmonton, Canada

**Keywords:** Cardiovascular medicine, Kinesiology, Respiratory medicine

## Abstract

We hypothesized that increased cardiorespiratory fitness (CRF) slows down a person’s aging, particularly in individuals with chronic airflow limitation (CAL). Participants aged ≥40 years (*n* = 78) had baseline blood DNA methylation profiled and underwent cardiopulmonary cycle exercise testing at baseline and at three years. Epigenetic clocks were calculated and tested for their association with CRF using linear regression. Differentially methylated genes associated with CRF were identified using a robust linear model. Higher CRF at baseline was associated with lower age acceleration in the epigenetic clocks DNAmAgeSkinBlood (*p = 0.016*), DNAmGrimAge (*p = 0.012*), and DNAmGrimAge2 (*p = 0.011*). These effects were consistent in individuals with CAL (DNAmGrimAge *p = 0.009* and DNAmGrimAge2 *p = 0.007*). CRF at three years was associated with baseline DNAmGrimAge (*p = 0.015*) and DNAmGrimAge2 (*p = 0.011*). Differentially methylated genes associated with CRF enriched multiple aging-related pathways, including cellular senescence. Enhancing CRF may be one intervention that can slow biological aging and improve health outcomes in chronic respiratory diseases.

## Highlights

• Younger epigenetic age is associated with cardiorespiratory fitness in airflow obstruction
• Self-reported activity and VO_2_ tests confirm the link between fitness and aging
• Cardiorespiratory fitness impacts DNA methylation along aging-related pathways

## Graphical abstract

**Figure:**
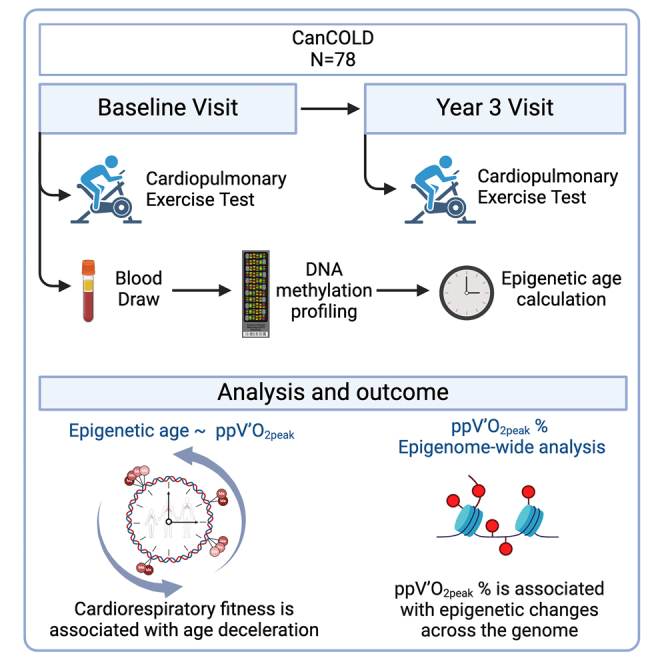

## Introduction

There were an estimated 703 million individuals aged 65 years or older globally in 2019 and projections suggest that this age group will reach 1.5 billion by 2050.^1^ With these demographic trends, diseases such as chronic obstructive pulmonary disease (COPD) will become increasingly prevalent, expected to affect 600 million patients by 2050.^2^ Interventions that promote healthier aging in patients with chronic lung diseases could significantly ease the burden that healthcare systems will face as the global population ages. One such intervention is to increase physical activity levels to improve cardiorespiratory fitness (CRF). Often quantified as the peak rate of oxygen consumption (V’O_2peak_) achieved during a cardiopulmonary exercise test (CPET), an individual’s CRF is directly related to the integrated function of cardiac, ventilatory, metabolic, and neuromuscular systems,^3^ thus reflecting their functional capacity. Epidemiological and clinical evidence suggests that CRF is a stronger predictor of mortality than risk factors such as smoking, hypertension, and obesity.^4^ It has been established that CRF is reduced in individuals with chronic airflow limitation (CAL)^5^ and that decreased exercise capacity is associated with mortality in patients with airflow obstruction.^6^

How we measure whether improvements in CRF can lead to healthier aging requires effective biomarkers that can capture an individual’s biological age, which in unhealthy populations could significantly diverge from their chronological age.^7^ Aging is the complex cumulative effect of many aberrant processes such as replicative senescence, genomic instability, cell-cell communication breakdown, stem cell exhaustion, mitochondrial dysfunction, loss of proteostasis, and DNA methylation (DNAm) disruptions.^8^ DNAm involves adding or removing a methyl group from a cytosine base next to a guanine base (CpG site), a process highly responsive to external stresses or exposures.^9^ Predictable DNAm changes that occur with age have facilitated the development of epigenetic clocks that can measure the difference between an individual’s estimated biological age and chronological age.^7^ Previous work has shown that high levels of self-reported physical activity levels are associated with slower epigenetic aging.^10^ Others have linked changes at specific CpG sites following physical exercise interventions, corresponding to genes involved in fat metabolism, cell growth, and neuronal differentiation.^11^^,^^12^ Objective assessments of the relationship between CRF (using V’O_2peak_) and epigenetic aging and epigenome-wide effects are still limited. To date, studies that have evaluated the relationship between epigenetic age and CRF as measured by the gold standard CPET have been limited to older male populations^13^ and skeletal muscle samples,^14^ while others have applied substitute measures of V’O_2_ using surrogates such as the Chester step test.^15^ Exploration of these relationships in individuals with impaired lung function has yet to be performed as well.

We hypothesized that people with higher baseline CRF would demonstrate a slower aging process as measured by the epigenetic clock and that this relationship is strongest in participants with CAL. We also hypothesized that epigenome-wide disruptions associated with CRF map to genes linked with aging. To investigate our hypotheses, we first determined if epigenetic age was associated with CPET-derived measures of V’O_2peak_. Next, we performed an epigenome-wide analysis first to assess the association of blood DNA methylation profiles with CRF and second, to identify gene-specific loci and related pathways epigenetically dysregulated in association with low CRF.

## Results

### Study cohort

The median age of participants was 56 years, 75% were females, 33% were current smokers, 21% were doctor-diagnosed with COPD, and 42% were doctor-diagnosed with asthma (Table 1). Overall, the caloric expenditure per week in exercise-related activities based on the Community Healthy Activities Model Program for Seniors (CHAMPS) score was 4,291 kilocalories/week. Table S1 shows the demographics of each subset within our study cohort. These two subsets were not statistically different in terms of V’O_2peak_ expressed as a percentage of the predicted normal value (ppV’O_2peak_) (*p = 0.430*).

**Table 1:** Study cohort demographic characteristics

Characteristic	Absolute	%Predicted
n (females, male)	59,19	–
Age, years	54.69 ± 7.42	–
Height, cm	167.27 ± 8.07	–
Body mass, kg	78.15 ± 17.30	–
BMI, kg/m^2^	27.84 ± 5.38	–
Smoking Status	–	–
Never, *n* (%)	41 (53)	
Former, *n* (%)	11 (14)	
Current, *n* (%)	26 (33)	
COPD, *n* (%)	18 (23)	–
Asthma, *n* (%)	33 (42)	–
Hypertension, *n* (%)	15 (19)	–
Type 1 diabetes, *n* (%)	1 (1)	–
Type 2 diabetes, *n* (%)	3 (4)	–
Post-bronchodilator FEV_1_, L	2.51 ± 0.84	82.5 ± 17.5
Post-bronchodilator FVC, L	3.76 ± 1.01	97.2 ± 14.4
Post-bronchodilator FEV_1_/FVC (%)	66.1 ± 9.6	–
CHAMPS, kcal/week	4,291 ± 2,864	–
V’O_2peak_, L/min	1.73 ± 0.67	90.6 ± 23.0
V’O_2peak_, mL/kg/min	22.12 ± 6.94	–
Power output, Watts	122.99 ± 52.97	88.9 ± 28.0
Respiratory exchange ratio at peak exercise	1.11 ± 0.10	–
Heart rate at peak exercise	142 ± 23	90 ± 13

Data are expressed as mean ± standard deviation. BMI, body mass index; FVC, forced vital capacity; FEV_1_, forced expiratory volume in 1 s; D_L_CO, diffusing capacity of the lung for carbon monoxide; V’O_2_, rate of oxygen consumption at the symptom-limited peak of incremental cardiopulmonary cycle exercise testing; CHAMPS, Community Healthy Activities Model Program for Seniors questionnaire. Chronic obstructive pulmonary disease (COPD), asthma, hypertension, and diabetes were defined as ever diagnosed by a physician according to participant self-report.

### CRF is associated with younger epigenetic age

We first investigated the relationship between CRF and epigenetic age at baseline. The DNAmAgeSkinBlood clock showed the highest correlation with chronological age in our dataset based on the Pearson’s correlation coefficient (R = 0.90, *p < 2.200 × 10*^*−16*^) followed by DNAmFitAge, DNAmAgeHannum, DNAmAge, DNAmPhenoAge, DNAmGrimAge, and DNAmGrimAge2 (Table 2).

**Table 2:** Epigenetic age Pearson’s correlation with chronological age and cardiorespiratory fitness estimated as the peak rate of oxygen consumption expressed as a percentage of the predicted normal value

Epigenetic clocks	Chronological age		ppV’O_2peak_	
	R	*p*	R	*p*
DNAmAge	0.82	*<2.200 × 10*^*−*^*^16^*	−0.25	*0.031*
DNAmAgeHannum	0.86	*<2.200 × 10*^*−*^*^16^*	−0.27	*0.016*
DNAmAgeSkinBlood	0.90	*<2.200 × 10*^*−*^*^16^*	−0.26	*0.024*
DNAmGrimAge	0.69	*2.042 × 10*^*−*^*^12^*	−0.37	*0.001*
DNAmGrimAge2	0.68	*1.004 × 10*^*−*^*^11^*	−0.37	*0.001*
DNAmPhenoAge	0.81	*<2.200 × 10*^*−*^*^16^*	−0.25	*0.026*
DNAmFitAge	0.87	*<2.200 × 10*^*−*^*^16^*	−0.31	*0.006*

Pearson’s correlation (R) and corresponding *p* value for the linear regression the between epigenetic clocks and chronological age, and ppV’O_2peak_ (unadjusted tests).

**Figure 1:**
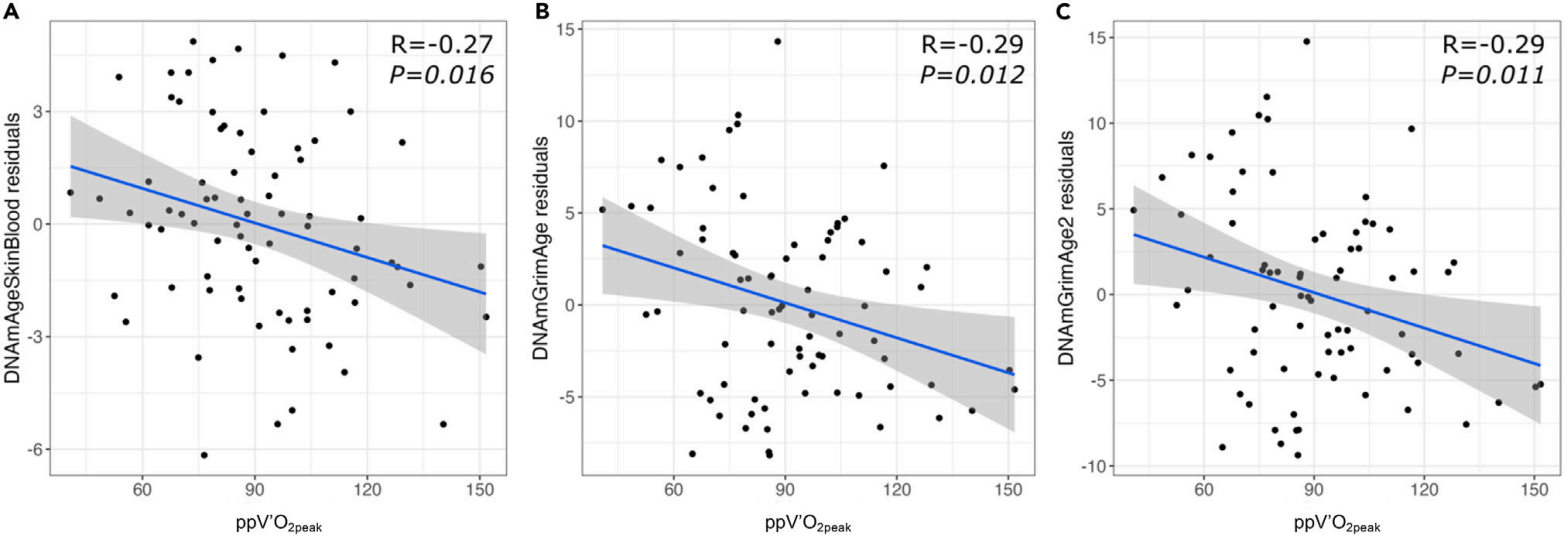
Higher peak oxygen consumption at three years is correlated with younger epigenetic age Linear relationship between epigenetic age residuals and the peak rate of oxygen consumption expressed as a percentage of the predicted normal value (ppV’O_2peak_) at baseline for (A) DNAmAgeSkinBlood, (B) DNAmGrimAge, and (C) DNAmGrimAge2. Gray shadow represents the standard error for the linear regression. Epigenetic age acceleration residuals (DNAmAgeSkinBlood residuals) used for these tests were obtained from the linear regression of epigenetic clocks on chronological age, sex, cigarette smoking status, CD8 T cells, CD4 T cells, NK cells, B cells, monocytes, and granulocytes. DNAmGrimAge and DNAmGrimAge2 age acceleration residuals used for these tests were obtained from the linear regression of epigenetic clocks on chronological age, sex, CD8 T cells, CD4 T cells, NK cells, B cells, monocytes, and granulocytes. Each epigenetic clock shown demonstrates that lower CRF is significantly associated with greater age acceleration. R corresponds to the Pearson’s correlation and meta-analysis *p* value for the linear relationship.

DNAmGrimAge (R = −0.37, *p = 0.001*) and DNAmGrimAge2 (R = −0.37, *p = 0.001*) showed the strongest correlations with ppV’O_2peak_ (Table 2). Our meta-analysis revealed that lower ppV’O_2peak_ was associated with higher epigenetic aging residuals (indicative of greater age acceleration) based on DNAmAgeSkinBlood (meta-analysis estimate = −0.030, *p = 0.016*), DNAmGrimAge (meta-analysis estimate = −0.061, *p = 0.012*), and DNAmGrimAge2 (meta-analysis estimate = −0.067, *p = 0.011*). The association between DNAmFitAge and ppV’O_2peak_ reached borderline significance (meta-analysis estimate = −0.003, *p = 0.050*). Figure 1 shows the correlation between epigenetic age acceleration residuals and ppV’O_2peak_ for each significant test. The direction of the effects identified in the meta-analyses presented here was consistent with subset-specific effects (Table S2); for instance, increased CRF in both subsets was associated with reduced DNAmGrimAge acceleration residuals. Additional analyses showed that lower absolute and relative V’O_2peak_ were associated with greater epigenetic age acceleration based on DNAmGrimAge ([absolute CRF] meta-analysis estimate = −4.55, *p = 0.004*; [relative CRF] meta-analysis estimate = −0.30, *p = 0.010*), DNAmGrimAge2 ([absolute CRF] meta-analysis estimate = −4.89, *p = 0.005*; [relative CRF] meta-analysis estimate = −0.34, *p = 0.007*), and DNAmAgeSkinBlood ([absolute CRF] meta-analysis estimate = −2.28, *p = 0.015*; [relative CRF] meta-analysis estimate = −0.17, *p = 0.005*) (Table S3). Similarly, we found that low self-reported physical activity (kilocalorie expenditure per week) using total CHAMPS scores was associated with epigenetic age acceleration (DNAmGrimAge meta-analysis estimate = −9.05 × 10^−4^, *p = 2.75* × *10*^*−04*^, DNAmGrimAge2 meta-analysis estimate = −8.26 × 10^−4^, *p = 2.29* × *10*^*−04*^) (Table S4).

### CRF and epigenetic age in airflow limitation

We further investigated if the relationship between ppV’O_2peak_ and DNAmGrimAge and DNAmGrimAge2 was maintained in people with CAL (*n* = 50). Both DNAmGrimAge (R = 0.42, *p = 0.003*) and DNAmGrimAge2 (R = 0.43, *p = 0.002*) showed significant inverse correlations with ppV’O_2peak_; furthermore, epigenetic age acceleration residuals were significantly associated with ppV’O_2peak_ in CAL following meta-analysis (DNAmGrimAge meta-analysis estimate = −0.075, *p = 0.009*, and DNAmGrimAge2 meta-analysis estimate = −0.084, *p = 0.007*). In individuals without CAL (*n* = 28), neither DNAmGrimAge (R = −0.21, *p = 0.278*) nor DNAmGrimAge2 (R = −0.19, *p = 0.326*) was significantly correlated with ppV’O_2peak_ (Table 3).

**Table 3:** Peak rate of oxygen consumption expressed as a percentage of the predicted normal value and epigenetic clocks in participants with or without chronic airway limitation

Group	Epigenetic clocks	R (*p value*)	Meta-analysis estimate	∗Meta-analysis *p* value
Chronic airflow limitation (*n* = 50)	DNAmGrimAge	−0.42 (*0.003*)	−0.075	*0.009*
	DNAmGrimAge2	−0.43 (*0.002*)	−0.084	*0.007*
No chronic airflow limitation (*n* = 28)	DNAmGrimAge	−0.21 (*0.174*)	−0.005	*0.775*
	DNAmGrimAge2	−0.19 (*0.326*)	−0.011	*0.598*

R (*p value*) – Pearson’s correlation and corresponding *p* value for the linear regression between the between epigenetic clocks and ppV’O_2peak_. Meta-analysis corresponds to the estimate and *p* value for the test of the effect of ppV’O_2peak_ on age acceleration residuals. Epigenetic age acceleration residuals used for these tests were obtained from the linear regression of epigenetic clocks on chronological age, sex, CD8 T cells, CD4 T cells, NK cells, B cells, monocytes, and granulocytes.

### Epigenetic age and CRF over three years

**Figure 2:**
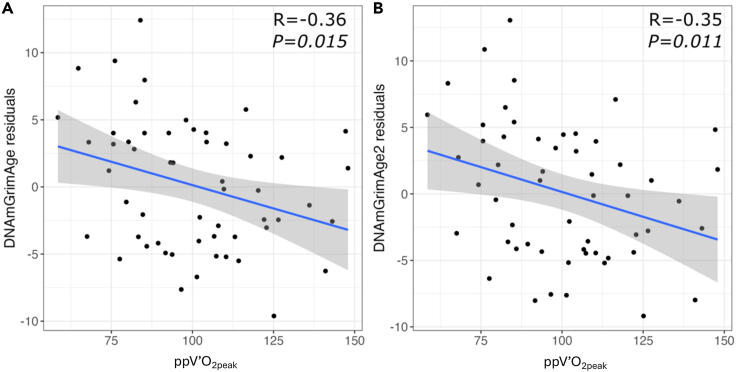
Higher peak oxygen consumption at baseline is correlated with younger epigenetic age Linear relationship between baseline epigenetic age residuals and the peak rate of oxygen consumption expressed as a percentage of the predicted normal value (ppV’O_2peak_) at three years for (A) DNAmGrimAge and (B) DNAmGrimAge2. Gray shadow represents the standard error for the linear regression. DNAmGrimAge and DNAmGrimAge2 residuals were adjusted for chronological age, sex, CD8 T cells, CD4 T cells, NK cells, B cells, monocytes, and granulocytes (epigenetic age acceleration residuals). Each epigenetic clock shown demonstrates that lower CRF at three years is significantly associated with greater age acceleration at baseline. R corresponds to Pearson’s correlation and p represents the meta-analysis *p* value for the linear relationship.

59 participants also performed CPETs approximately three years after their baseline visit (median follow-up: 38 months [interquartile range 36–40 months]). Absolute and relative V’O_2peak_ stayed consistent over the three-year period (absolute V’O_2peak_ at baseline 1.73 ± 0.67 versus absolute V’O_2peak_ three years after baseline 1.85 ± 0.66 L/min [*p* = 0.30]; relative V’O_2peak_ 22.12 ± 6.95 versus relative V’O_2peak_ three years after baseline 23.47 ± 6.57 mL/kg/min [*p* = 0.19]). Epigenetic age acceleration residuals at baseline were still associated with ppV’O_2peak_ at three years, demonstrating significant correlations with DNAmGrimAge (R = −0.36, *p = 0.015*) and DNAmGrimAge2 (R = −0.35, *p = 0.011*) (Figure 2). Furthermore, epigenetic age acceleration residuals and ppV’O_2peak_ were significantly associated (DNAmGrimAge meta-analysis estimate = −0.08, *p = 0.015*, and DNAmGrimAge2 meta-analysis estimate = −0.09, *p = 0.011*). Greater epigenetic age acceleration residuals at baseline based on DNAmGrimAge ([absolute CRF] meta-analysis estimate = −7.33, *p = 0.006*; [relative CRF] meta-analysis estimate = −0.60, *p = 1.533* × *10*^*−04*^) and DNAmGrimAge2 ([absolute CRF] meta-analysis estimate = −5.97, *p = 0.005*; [relative CRF] meta-analysis estimate = −0.64, *p = 3.731* × *10*^*−04*^) were also associated with decreased absolute and relative V’O_2peak_ at three years (Table S3). No significant relationship was found between epigenetic age at baseline and change in V’O_2peak_ over three years, however.

### CRF may influence the blood epigenome

**Figure 3:**
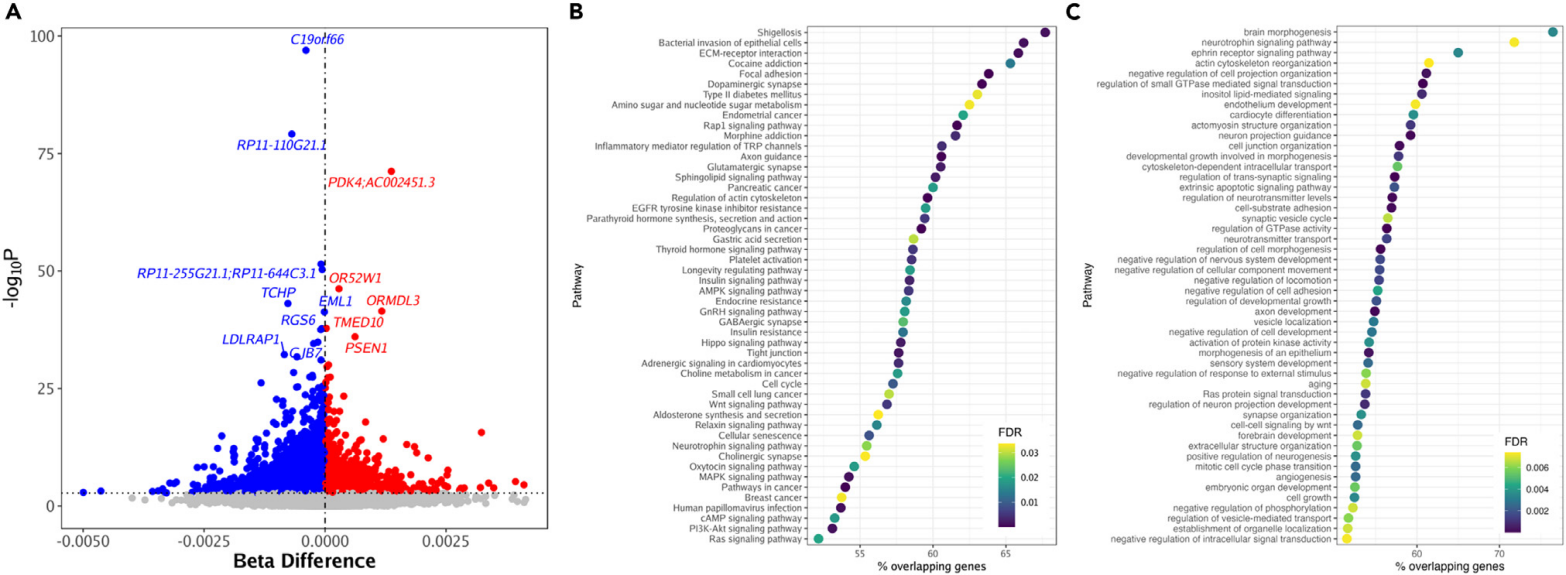
Differentially methylated sites and pathways associated with the peak rate of oxygen consumption expressed as a percentage of the predicted normal value (A) The x axis represents the robust linear model (rlm) estimated effects on the methylation beta-values, the y axis represents the rlm −log10 *p* value for M-values. For each 1% increase in ppV’O2peak, DNA methylation decreases (hypomethylation = blue) or increases (hypermethylation = red). (B) The top 50 enriched KEGG pathways associated with ppV’O_2peak_. Color intensity (black to yellow) represents the level of significance. (C) The top 50 enriched GO pathways associated with ppV’O_2peak_.

Our epigenome-wide blood DNA methylation analysis yielded 23,253 differentially methylated positions (DMPs) significantly associated with ppV’O_2peak_ (Figure 3A; Table S5), corresponding to 12,115 unique genes. Top DMPs based on statistical significance (false discovery rate [FDR]) corresponded to both protein-coding and non-protein-coding genes (*C19orf66*, *RP11-110G21.1*, *PDK4*, *AC002451.3*, *RP11-255G21.1*, *RP11-644C3.1*, and *TCHP*). Our enrichment analysis showed that differentially methylated genes associated with ppV’O_2peak_ were enriched for 62 Kyoto Encyclopedia of Genes and Genomes (KEGG) pathways (Table S6) and 154 Gene Ontology (GO) pathways (Table S7). These include numerous aging-related pathways such as Ras signaling, aging, longevity regulation, cellular senescence, insulin resistance, and PI3K-Akt, AMPK, and Rap1 signaling. The top 50 KEGG pathways are shown in Figure 3B and the top 50 GO pathways are shown in Figure 3C.

## Discussion

Our analyses on DNA methylation and CPET-derived CRF show that higher CRF as measured by ppV’O_2peak_ was associated with younger blood epigenetic age particularly in individuals with CAL. Second, using longitudinal CPET data, we showed that blood epigenetic age at baseline captures future CRF over a three-year period. Finally, our epigenome-wide differential blood DNA methylation analysis suggests that CRF influences the epigenetic regulation of numerous aging-related biological pathways. Together, these findings indicate that CRF is a modifiable exposure that contributes to healthy aging through epigenetic regulation.

The V’O_2peak_ on CPET declines with age by approximately 10% per decade in sedentary individuals older than 25 years, and by about 15% per decade between the ages of 50 and 75.^16^ This decline can be aggravated by exposures such as cigarette smoking^17^ and will impact an individual’s ability to perform routine physical activity, including exercise. On the other hand, previous work on professional athletes has shown that healthy aging may be achievable through lifelong physical activity.^18^ In our cohort, participants with the lowest CRF appeared to be biologically older, raising the tantalizing possibility that interventions that can improve an individual’s V’O_2peak_ could potentially slow their aging process. Although none have used the gold standard CPET as a measure of CRF, other investigators have implemented exercise programs in select patient populations to show benefits in epigenetic age. For instance, in older adults with myeloid malignancies, Loh et al. used a mobile health exercise intervention to show that improvements in 6-min walk distance and handgrip strength were associated with decreases in blood DNAmGrimAge and DNAmPhenoAge.^19^ Similarly, a small trial of eight weeks of physical training in 18 sedentary women showed significant attenuation of epigenetic age acceleration for those women with the highest blood epigenetic ages at baseline.^20^ Given that blood epigenetic age is strongly associated with mortality in the general population,^21^ these small yet significant changes could result in meaningful improvements in health outcomes.

Although Kawamura et al. previously showed in older Japanese men that relative V’O_2peak_ was negatively associated with blood DNAmGrimAge,^13^ we extend these findings by demonstrating a similar effect in a population that included females and further that these associations are stable over time. Specifically, we found that baseline blood epigenetic age was associated not just with concurrent CRF but also CRF at a three-year interval, suggesting that epigenetic age may predict longitudinal changes in ppV’O_2peak_ if tested in a larger study cohort. Interestingly, the stability of the association between epigenetic age and ppV’O_2peak_ over time appeared to be limited to two clocks specifically: DNAmGrimAge and DNAmGrimAge2 (both of which demonstrated the strongest correlations with ppV’O_2peak_ at baseline as well). These two clocks outperformed even DNAmFitAge, which was specifically trained on measures of V’O_2_ for its derivation.^22^ It is not clear why some epigenetic clocks were able to capture CRF better than others. Both versions of DNAmGrimAge are not only biomarkers for aging but also biomarkers for mortality risk that included inflammatory proteins and tobacco smoking in their derivations.^21^^,^^23^ Our group has previously shown that the correlation between blood and airway epithelial epigenetic age is strongest when using DNAmGrimAge compared to the other epigenetic clocks and thus may reflect a lung-intrinsic aging process the best.^24^ As such, DNAmGrimAge may be particularly effective at capturing the contribution of the pulmonary system to low levels of CRF. Primary cell culture work performed by other investigators has shown that the specific biological aging processes that are exhibited in the epigenetic clock are mitochondrial dysfunction and nutrient sensing,^25^ both playing pivotal roles in exercise tolerance.

Our epigenome-wide differential analysis suggests that ppV’O_2peak_ is highly associated with thousands of DNA methylation changes across the genome. For instance, *PDK4* was highlighted as a top differentially methylated gene associated with CRF. Previous work has also found that CpGs within *PDK4* are modulated by exercise in muscle cells^26^; the same research also reported that other genes highlighted by our work, such as *PPARGC1A* (*FDR = 0.016*), are associated with physical activity.^26^ *PDK4* is a metabolic gene whose transcription of a mitochondrial protein in skeletal muscle is upregulated in response to exercise, particularly in muscles with low glycogen content.^27^ *PPARGC1A* encodes a transcriptional coactivator integral in energy metabolism. Through nitric oxide-mediated pathways, physical exercise increases *PPARGC1A* transcription, which then promotes oxidative phosphorylation in muscles to adapt to endurance training.^28^ Together, these findings consistently show the involvement of differentially methylated genes within metabolic pathways that are essential for the ability to perform muscular work and, by extension, maintaining or improving CRF.

### Limitations of the study

Our investigation has implicated DNA methylation as a mechanistic link behind the effects of CRF on aging and other biological processes; however, our analysis has limitations. First, our sample size was limited and thus our findings may not reflect all age groups (notably younger individuals) in the general population. Nevertheless, these findings may be important to middle- and older-aged adults. The sample size also limited our ability to understand the relationship between epigenetic age and ppV’O_2peak_ in specific disease groups such as asthma or by variable cigarette smoking exposure. Interactions between CRF, sex, and CAL status would also need to be performed. Future replication in a larger cohort should address these questions. Second, the downstream effects of epigenetic changes linked to CRF were not explored by our research as we lacked concurrent mRNA or protein readouts. Third, although our tests suggest that epigenetic age can capture future ppV’O_2peak_, we were likely underpowered to accurately assess the relationship between epigenetic age and change in CRF. Whether the epigenetic clock can truly serve as a predictive biomarker identifying those at greatest risk for a decline in CRF needs to be evaluated in a larger cohort. Finally, we lacked an exercise intervention to test the hypothesis that improved ppV’O_2peak_ could reduce epigenetic aging.

Notwithstanding the limitations of this study, we have shown here that CRF may contribute to slowing biological aging, particularly in individuals with CAL, and that DNA methylation may be partly responsible for the genome-wide consequences of CRF beyond aging. These findings suggest that epigenetic clocks could be developed as biomarkers for CRF and emphasize the importance of maintaining a physically active lifestyle and optimal CRF levels to promote healthy aging.

## Resource availability

### Lead contact

Further information and requests for resources should be directed to and will be fulfilled by the lead contact, Dr. Janice Leung (Janice.Leung@hli.ubc.ca).

### Materials availability

Data from the CanCOLD study are available for researchers upon request to access via https://cancold.ca/. Methylation data have been deposited into GEO: GSE255929.

### Data and code availability

• Data: Data from the CanCOLD study are available for researchers upon request to access via https://cancold.ca/. Methylation data have been deposited into GEO: GSE255929.
• Code: This paper does not report original code.
• Additional information including R code required to reanalyze the data reported in this paper is available from the corresponding author upon request.

## Acknowledgments

The authors thank the people who participated in the study and the many members of the CanCOLD collaborative research group: Executive Committee: J.B. (10.13039/100008582McGill University, Montreal, QC, Canada); Wan C Tan, J Mark FitzGerald, and D.D.S. (10.13039/501100005247University of British Columbia, Vancouver, BC, Canada); Darcy D Marciniuk (10.13039/100008920University of Saskatchewan, Saskatoon, SK, Canada); Denis E O'Donnell (10.13039/501100003321Queen's University, Kingston, ON, Canada); Paul Hernandez (10.13039/501100002977Dalhousie University, Halifax, NS, Canada); Kenneth R Chapman (10.13039/501100003579University of Toronto, Toronto, ON, Canada); Brandie Walker (10.13039/100008459University of Calgary, Calgary, AB, Canada); Shawn Aaron (10.13039/100008572University of Ottawa, Ottawa, ON, Canada); and François Maltais (University of Laval, Quebec City, QC, Canada). International Advisory Board: Jonathon Samet (the Keck School of Medicine of USC, California, USA), Milo Puhan (John Hopkins School of Public Health, Baltimore, USA), Qutayba Hamid (10.13039/100008582McGill University, Montreal, QC, Canada), and James C Hogg (10.13039/501100005247University of British Columbia, Vancouver, BC, Canada). Operations Center: J.B. (Principal Investigator), D.D., Palmina Mancino, Pei Zhi Li, Dennis Jensen, and Carolyn Baglole (10.13039/100008582McGill University, Montreal, QC, Canada); Yvan Fortier (Laboratoire telematique, Quebec Respiratory Health Network, 10.13039/501100000156Fonds de la recherche en sante du Quebec); Wan C Tan (co-Principal Investigator), D.D.S., Julia Yang, Jeremy Road, Joe Comeau, Adrian Png, Kyle Johnson, Harvey Coxson, Jonathon Leipsic, and Cameron Hague (10.13039/501100005247University of British Columbia, Vancouver, BC, Canada); and Miranda Kirby (10.13039/100007891Ryerson University, Toronto, ON, Canada) Economic Core: Mohsen Sadatsafavi (10.13039/501100005247University of British Columbia, Vancouver, BC, Canada). Public Health Core: Teresa To and Andrea Gershon (10.13039/501100003579University of Toronto, Toronto, ON, Canada). Data management and Quality Control: Wan C Tan and Harvey Coxson (10.13039/501100005247University of British Columbia, Vancouver, BC, Canada); J.B., Pei-Zhi Li, Zhi Song, Andrea Benedetti, and D.J. (10.13039/100008582McGill University, Montreal, QC, Canada); Yvan Fortier (Laboratoire telematique, Quebec Respiratory Health Network, FRQS); and Miranda Kirby (10.13039/100007891Ryerson University, Toronto, ON, Canada). Field Centers: Wan C Tan (Principal Investigator), Christine Lo, Sarah Cheng, Elena Un, Cynthia Fung, Wen Tiang Wang, Liyun Zheng, Faize Faroon, Olga Radivojevic, Sally Chung, and Carl Zou (10.13039/501100005247University of British Columbia, Vancouver, BC, Canada); J.B. (Principal Investigator), Palmina Mancino, Jacinthe Baril, and Laura Labonte (10.13039/100008582McGill University, Montreal, QC, Canada); Kenneth Chapman (Principal Investigator), Patricia McClean, and Nadeen Audisho (10.13039/501100003579University of Toronto, Toronto, ON, Canada); Brandie Walker (Principal Investigator), Curtis Dumonceaux, and Lisette Machado (10.13039/100008459University of Calgary, Calgary, AB, Canada); Paul Hernandez (Principal Investigator), Scott Fulton, Kristen Osterling, and Denise Wigerius (University of Halifax, Halifax, NS, Canada); Shawn Aaron (Principal Investigator), Kathy Vandemheen, Gay Pratt, and Amanda Bergeron (10.13039/100008572University of Ottawa, Ottawa, ON, Canada); Denis O'Donnell (Principal Investigator), Matthew McNeil, and Kate Whelan (10.13039/501100003321Queen's University, Kingston, ON, Canada); François Maltais (Principal Investigator) and Cynthia Brouillard (University of Laval, Quebec City, QC, Canada); Darcy Marciniuk (Principal Investigator), Ron Clemens, Janet Baran, and Candice Leuschen (10.13039/100008920University of Saskatchewan, Saskatoon, SK, Canada).

The Canadian Cohort Obstructive Lung Disease (CanCOLD; NCT00920348) study is currently funded by the 10.13039/501100021743Canadian Respiratory Research Network and industry partners 10.13039/100008207AstraZeneca Canada, Boehringer Ingelheim Canada Ltd, 10.13039/100004330GlaxoSmithKline Canada Ltd, and 10.13039/100004336Novartis. This work was supported with funding from the 10.13039/501100000024Canadian Institutes of Health Research. Funding sources had no role in the writing of the manuscript or the decision to submit for publication. Authors were not paid to write this article by any company or agency. Authors were not precluded from accessing data in the study and accept responsibility to submit for publication. A.I.H.C. is supported by the Michael Smith Health Research BC Trainee Award. M.S.K., G.J.K., D.D.S., D.J., and J.M.L. are supported by the Canada Research Chairs program.

CanCOLD is funded by the 10.13039/501100021743Canadian Respiratory Research Network, 10.13039/100008207AstraZeneca Canada, Boehringer Ingelheim Canada Ltd, GlaxoSmithKline Canada Ltd, and 10.13039/100004336Novartis. This work was supported by the 10.13039/501100000024Canadian Institutes of Health Research.

## Author contributions

J.M.L., A.I.H.C., and C.P. wrote the manuscript draft. A.I.H.C., X.L., and C.X.Y. processed the data and conducted the statistical analyses. G.J.K. and G.J.F. contributed to data analysis and interpretation. C.P., D.D., W.T., J.B., D.J., D.D.S., G.J.K., and M.K.S. contributed to the CPET data acquisition and analyses. Q.D. and J.M.L. designed the study. A.A., J.L.M., and M.S.K. profiled the samples for DNA methylation. D.D., J.B., W.T., and D.D.S. generated the data used for this study. The CanCOLD Collaborative Research Group provided access to samples. All authors revised and approved the final manuscript.

## Declaration of interests

The authors declare no competing interests.

## STAR★Methods

### Key resources table

**Table:** 

REAGENT or RESOURCE	SOURCE	IDENTIFIER
**Biological samples**		

Peripheral blood samples	ClinicalTrials.gov	NCT00920348

**Critical commercial assays**		

Illumina Infinium MethylationEPIC BeadChip microarray	Illumina	N/A

**Deposited data**		

DNA methylation data	This paper	GEO: GSE255929

**Software and algorithms**		

ChAMP	R statistical software	Tian et al.^29^
minfi	R statistical software	Aryee et al.^30^
limma	R statistical software	Ritchie et al.^31^
Clock Foundation tool	Clock Foundation	https://dnamage.clockfoundation.org
WebGestaltR	R statistical software	Liao et al.^32^
metafor	R statistical software	Viechtbauer^33^

### Experimental model and study participant details

#### Human participants

• Age: 54.69 ± 7.42 years
• Females *n* = 59, Males *n* = 19
• Sex effects were not investigated due to batch effects
• Peripheral blood samples

### Method details

#### Study cohort

The Canadian Cohort of Obstructive Lung Disease (CanCOLD) is a prospective cohort study.^34^ Briefly, the CanCOLD cohort (*n* = 1561 participants) was created by sampling all participants with chronic obstructive pulmonary disease (COPD) from the Canadian Obstructive Lung Disease (COLD) study and an equal number of age- and sex-matched peers without COPD from the same study (ClinicalTrials.gov identifier NCT00920348, institutional ethics approval provided at each study site). The CanCOLD study included individuals aged ≥40 years representing nine urban and suburban areas in Canada (Vancouver, Saskatoon, Calgary, Toronto, Ottawa, Kingston, Montreal, Quebec City, and Halifax). For the present work, we used a subset of 78 participants with peripheral blood samples and CPET records collected at baseline and at three years of follow-up. These participants were previously profiled by two individual research teams (subset 1: *n* = 30; subset 2: *n* = 48). Clinical and demographic characteristics of the study cohort are shown in Table 1. Specific clinical and demographic characteristics by each subset are provided in Table S1.

A summary of the study workflow is presented in Figure S1.

#### CPET measurements

To determine CRF (i.e., peak oxygen uptake or V’O_2peak_), CPETs were performed at baseline and at three years on an electronically braked cycle ergometer with the use of a computerized CPET system (Vmax, SensorMedics [seven sites]; TrueOne, Parvomedics [one site]; Ergocard, Medisoft [one site]). The CPET protocol was standardized across sites and consisted of a steady-state rest period of three to 10 min, 1 min of unloaded pedaling, and then a 10W increase in power output every minute, starting at 10W, until one of the following termination criteria occurred: (1) limited by symptoms (i.e., unwilling to continue exercising because of the discomfort associated with the exercise); OR (ii) unable to maintain a frequency of at least 40 rpm despite continued encouragement to increase the frequency to 50–70 rpm; OR (iii) unable to continue safely (in the opinion of the supervising technician). Gas exchange and breathing pattern variables were collected with participants breathing through a mouthpiece and low resistance flow transducer. Nasal passages were occluded with a nose clip. The rate of oxygen consumption at peak exercise (V’O_2peak_) was taken as the V’O_2_ averaged over the last 30-s of loaded pedaling and expressed as a percentage of the predicted normal V’O_2peak_ (ppV’O_2peak_) using the reference equation published by Lewthwaite et al.,^35^ which takes into account sex, height, body mass, and chronological age and is based on the 50^th^ percentile of the CanCOLD cohort. The main focus of our analyses was on ppV’O_2peak_.

In addition, the Community Healthy Activities Model Program for Seniors (CHAMPS) questionnaire was administered to participants to determine self-reported physical activity.^36^ Energy expenditure was estimated based on CHAMPS according to the American College of Sports Medicine equation.^37^

#### DNA methylation

Peripheral blood samples were collected from participants at baseline and DNA was extracted from the buffy coat. DNA methylation profiles were obtained using the Illumina Infinium MethylationEPIC BeadChip microarray, which interrogates 863,904 DNA methylation sites (CpG probes) across the genome. The samples were profiled at two laboratories (subset 1: *n* = 30; subset 2: *n* = 48). Raw data were processed separately using filtering, quality controls and normalization steps according to methods^29^^,^^30^ that have been standardized by our laboratory.^24^

#### Epigenetic age and epigenome-wide analysis

DNA methylation profiles were inputted into the Clock Foundation tool (https://dnamage.clockfoundation.org) to obtain epigenetic age estimates based on multiple epigenetic clocks, including DNAmAge,^7^ DNAmSkinBlood,^38^ DNAmAgeHannum,^39^ DNAmGrimAge,^21^ and DNAmPhenoAge,^40^ DNAmGrimAge2,^23^ and DNAmFitAge.^22^ Epigenetic age acceleration is interpreted as follows: a positive epigenetic age residual resulting from the regression of epigenetic age on chronologic age indicates faster age acceleration. First, we obtained epigenetic age acceleration residuals based on the following equation:

Epigeneticage∼Chronologicalage+Sex+Cigarettesmokingstatus+CD8Tcells+CD4Tcells+NKcells+Bcells+Monocytes+Granulocytes

Covariates were chosen as cell type proportion, sex, and smoking status are known to affect DNA methylation.^41^ Cell type distribution inferences for each blood sample were calculated according to methods by Houseman et al.^42^ using the Clock Foundation tool. Briefly, the Houseman method infers the distribution of white blood cells from a reference set of DNA methylation signatures derived from purified leukocyte samples. Cigarette smoking was defined as “current,” “former,” or “never” smoking at the baseline visit. This analysis was conducted for each epigenetic clock and subset separately. For two specific clocks, the model was modified: first, for DNAmPhenoAge, cell counts were not included because inflammatory cells are main predictors included in this clock’s calculation^40^; second, for DNAmGrimAge^21^ and DNAmGrimAge2,^23^ cigarette smoking was not included as this is a main predictor of these two clocks. In addition, subset 2 only included females (Table S1), thus sex was not included in its analysis.

We then used a univariate model to test the association between the epigenetic age acceleration residual with ppV’O_2peak_ using the equation shown below:

$$Epigenetic\;age\;acceleration\;residual\;∼\;ppV’O_{2peak}$$

We later combined the subset findings for each clock using a meta-analysis implemented in the R package metafor (fixed-effects model); this approach was used because of the demographic differences between the two subsets and technical differences in the DNA methylation profiling. The associations between epigenetic age acceleration residuals and absolute V’O_2peak_ (L/min) and relative V’O_2peak_ (mL/kg/min) and CRF were also tested. Significant associations were defined at *p < 0.05*.

Given that lung function is associated with CRF,^5^ we tested if the relationship between epigenetic age and CRF was consistent in individuals with chronic airflow limitation (CAL) within our study cohort. CAL was defined as a post-bronchodilator forced expiratory volume in 1 s to forced vital capacity (FEV_1_/FVC) ratio less than the lower limit of normal (LLN).^43^ We next repeated these analyses using baseline epigenetic age acceleration residuals and measurements of ppV’O_2peak_ taken three years after baseline (*n* = 59). We determined whether participant self-reported physical activity was also associated with epigenetic age using energy expenditure as determined by CHAMPS scores by the same approach described above.

After log transforming methylation beta values to M values, we then conducted a genome-wide differential DNA methylation analysis using the following robust linear model^31^:

$${M\;value=Chronological\;age+Sex+Cigarette\;smoking\;status+ppV’O}_{2peak}+PlasmaBlasts+CD4T\;cells+NK\;cells+Granulocytes+PC1+PC2$$

To select the covariates included in the model we conducted principal component analysis (PCA) based on DNA methylation by each subset and used the first two principal components (PC) to assess the effect of potential covariates on methylation (Figures S2 and S3). Since subset 2 only included females, sex was not included in its analysis. We later combined the subset findings using the R package metafor (fixed-effects model).^33^ We defined significant results based on a significant meta-analysis association at a False Discovery Rate (FDR) < 0.05 and consistent effects direction (Beta Fold Change [BetaFC]) in both individual analyses and the meta-analysis. These DMPs were used for downstream analysis. All statistical analyses were conducted in R software (version 4.3.1).

#### Enrichment analyses

We identified Kyoto Encyclopedia of Genes and Genomes (KEGG) and Gene Ontology pathways that were significantly (FDR<0.05) enriched by genes that corresponded to DMPs associated with ppV’O_2peak_ using the R package WebGestaltR.^32^

### Quantification and statistical analysis

#### Statistical analysis and software (STAR methods)

• R statistical software
  ◦ ChAMP^29^
  ◦ Minfi^30^
  ◦ Limma^31^
  ◦ WebGGestaltR^32^
  ◦ Metafor^33^
• Clock Foundation tool (STAR Methods)
  ◦ https://dnamage.clockfoundation.org

#### Statistical details

• n values (participants) (STAR Methods and results section)
  ◦ 78 Baseline CPET observations
    ▪ 59 (3-year visit)
  ◦ 78 DNA methylation profiles
• Definitions (STAR Methods)
  ◦ Cardiopulmonary exercise test (CPET)
  ◦ Peak rate of oxygen consumption V’O_2peak_
• V’O_2peak_ equation (STAR Methods)
  ◦ ${VO}_{2}=\left(0.012905699\times Height\left[cm\right]\right)+(0.007291081\times Weight\left[kg\right]+\left(0.564188602\times Sex\left[0=female,1=male\right]\right)-\left(0.016521850\times Age\left[years\right]\right)-\;0.07607206$
• Robust linear model equation (STAR Methods)
  ◦ $M\;value=Chronological\;age+Sex+Cigarette\;smoking\;status+{VO}_{2}\;\%predicted+PlasmaBlasts+CD4T\;cells+NK\;cells+Granulocytes+PC1+P$
• Definition of center, and dispersion and precision measures
  ◦ Mean and standard deviation

#### Significance definition

• Significance Measures (STAR methods and figures)
  ◦ *P*-value <0.05
  ◦ False discovery rate (FDR<0.05)

## Supplemental information

Document S1. Figures S1–S3 and Data S1

Table S1. Subsets of CanCOLD participants and demographic overview, related to Table 1

Table S2. Significant ppV'O2peak – epigenetic age associations in meta-analysis, related to Figure 1

Table S3. Meta-analysis of epigenetic age [baseline] and absolute and relative VO2 [baseline and 3 years follow-up] in CanCOLD, related to Table 2

Table S4. Meta-analysis of epigenetic age [baseline] and CHAMPS [self-reported physical activity] [baseline] in CanCOLD, related to Table 2

Table S5. Differential methylation associated to ppV'O2peak, related to Figure 3

Table S6. KEGG pathways associated to ppV'O2peak differential methylation, related to Figure 3

Table S7. GO pathways associated to ppV'O2peak differential methylation, related to Figure 3
