# Supplementary material for: Younger epigenetic age is associated with higher cardiorespiratory fitness in individuals with airflow limitation
Source: iScience. 2024 Sep 13;27(10):110934. doi: 10.1016/j.isci.2024.110934 (PMC11465153; doi:10.1016/j.isci.2024.110934)

iScience, Volume *27*

Supplemental information

Younger epigenetic age is associated with higher cardiorespiratory fitness in individuals with airflow limitation

Ana I. Hernandez Cordero, Carli Peters, Xuan Li, Chen Xi Yang, Amirthagowri Ambalavanan, Julie L. MacIsaac, Michael S. Kobor, Gregory J. Fonseca, Dany Doiron, Wan Tan, Jean Bourbeau, Dennis Jensen, Don D. Sin, Graeme J. Koelwyn, Michael K. Stickland, Qingling Duan, Janice M. Leung, and the CanCOLD Collaborative Research Group

Data S1 section:

Data S1. STROBE checklist, related to main manuscript

STROBE Statement—Checklist of items that should be included in reports of ***cohort studies***

	Item No	Recommendation
**Title and abstract**	1	(*a*) Indicate the study's design with a commonly used term in the title or the abstract **-> Line 1 - 2**
		(*b*) Provide in the abstract an informative and balanced summary of what was done and what was found **-> Line 49 - 66**
**Introduction**		
Background/rationale	2	Explain the scientific background and rationale for the investigation being reported **-> Line 85 -118**
Objectives	3	State specific objectives, including any prespecified hypotheses **-> Line 120 - 126**
**Methods**		
Study design	4	Present key elements of study design early in the paper **-> Line 419 – 431**
Setting	5	Describe the setting, locations, and relevant dates, including periods of recruitment, exposure, follow-up, and data collection **-> Line 419 – 431**
Participants	6	(*a*) Give the eligibility criteria, and the sources and methods of selection of participants. Describe methods of follow-up. **-> Line 419 – 431**
		(*b*) For matched studies, give matching criteria and number of exposed and unexposed **n/a**
Variables	7	Clearly define all outcomes, exposures, predictors, potential confounders, and effect modifiers. Give diagnostic criteria, if applicable **-> Line 435 - 462**
Data sources/ measurement	8*	For each variable of interest, give sources of data and details of methods of assessment (measurement). Describe comparability of assessment methods if there is more than one group **-> Line 435 – 462**
Bias	9	Describe any efforts to address potential sources of bias **-> Line 464 - 486**
Study size	10	Explain how the study size was arrived at **-> Line 420 – 431**
Quantitative variables	11	Explain how quantitative variables were handled in the analyses. If applicable, describe which groupings were chosen and why **-> Line 464-521**
Statistical methods	12	(*a*) Describe all statistical methods, including those used to control for confounding**-> Line 529 - 561**
		(*b*) Describe any methods used to examine subgroups and interactions **-> Line 499 - 506**
		(*c*) Explain how missing data were addressed **n/a**
		(*d*) If applicable, explain how loss to follow-up was addressed **173- 175**
		(*e*) Describe any sensitivity analyses **n/a**
**Results**		
Participants	13*	(a) Report numbers of individuals at each stage of study—eg numbers potentially eligible, examined for eligibility, confirmed eligible, included in the study, completing follow-up, and analysed **-> Line130-135, 173-175, Table 1**

		(b) Give reasons for non-participation at each stage **n/a**
		(c) Consider use of a flow diagram **-> Line 433**
Descriptive data	14*	(a) Give characteristics of study participants (eg demographic, clinical, social) and information on exposures and potential confounders **-> Line130-135**
		(b) Indicate number of participants with missing data for each variable of interest **n/a**
		(c) Summarise follow-up time (eg, average and total amount) **-> Line 173- 175**
Outcome data	15*	Report numbers of outcome events or summary measures over time **-> Table 1, Line 173- 175**
Main results	16	(*a*) Give unadjusted estimates and, if applicable, confounder-adjusted estimates and their precision (eg, 95% confidence interval). Make clear which confounders were adjusted for and why they were included
		(*b*) Report category boundaries when continuous variables were categorized **-> Table 1**
		(*c*) If relevant, consider translating estimates of relative risk into absolute risk for a meaningful time period **n/a**
Other analyses	17	Report other analyses done—eg analyses of subgroups and interactions, and sensitivity analyses **-> Line 173- 175**
**Discussion**		
Key results	18	Summarise key results with reference to study objectives **-> Line 203 - 209**
Limitations	19	Discuss limitations of the study, taking into account sources of potential bias or imprecision. Discuss both direction and magnitude of any potential bias **-> Line 260 - 279**
Interpretation	20	Give a cautious overall interpretation of results considering objectives, limitations, multiplicity of analyses, results from similar studies, and other relevant evidence **-> Line 276 -280**
Generalisability	21	Discuss the generalisability (external validity) of the study results **-> Line 260 - 279**
**Other information**		
Funding	22	Give the source of funding and the role of the funders for the present study and, if applicable, for the original study on which the present article is based **-> Line 320 -327**

Figure S1. Study overview, Related to Methods

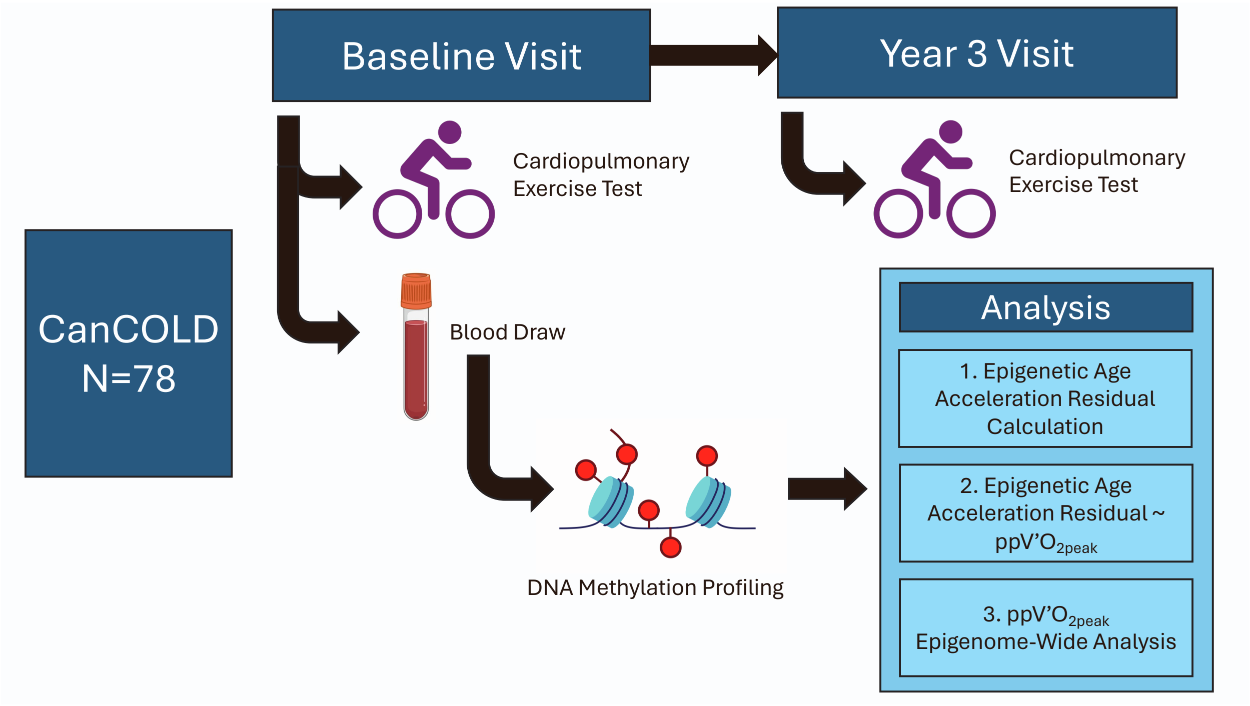

Figure S2. Covariate selection for meta-analysis (Subset 1**),** Related to STAR Methods

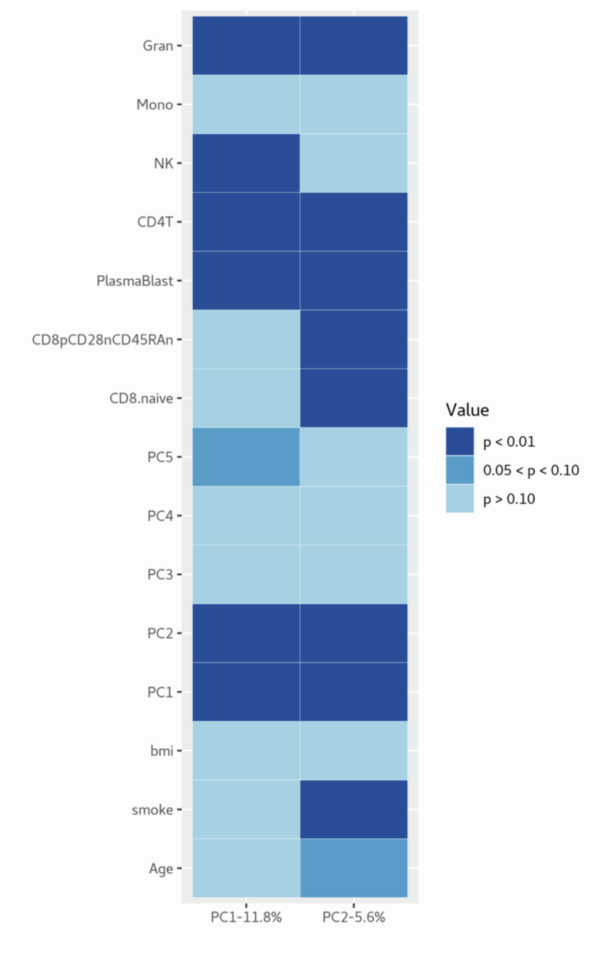

Figure S3. Covariate selection for meta-analysis (Subset 2**),** Related to STAR Methods

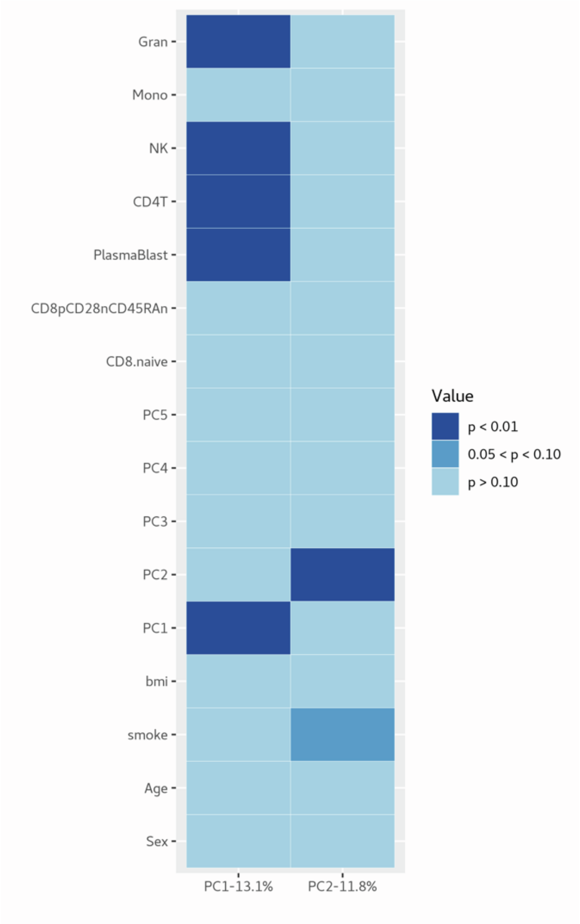

Supplement: Document S1. Figures S1–S3 and Data S1 [file mmc1.pdf]
